# Static posturography in addicted to illicit drugs and alcohol

**DOI:** 10.5935/1808-8694.20120015

**Published:** 2015-11-20

**Authors:** Daniela Affonso Moreira, Maurício Malavasi Ganança, Heloisa Helena Caovilla

**Affiliations:** a MSc in Sciences - Graduate Program in Human Communication Disorders - Federal University of São Paulo - Paulista School of Medicine (UNIFESP-EPM). Speech and Hearing Therapist; b Full Professor of Otorhinolaryngology - UNIFESP-EPM; c Senior Associate Professor of Otology and Neurotology - UNIFESP-EPM. Escola Paulista de Medicina (UNIFESP-EPM)

**Keywords:** alcoholism, *crack* cocaine, dizziness, ethanol, postural balance

## Abstract

The use of illicit drugs and alcohol can affect body balance.

**Aim:**

To evaluate balance control with static posturography in individuals addicted to illicit drugs, with or without alcohol abuse. Study design: Case-control, prospective.

**Methods:**

47 users of illicit drugs, with or without alcohol abuse, and a homogeneous control group consisting of 47 healthy individuals were submitted to a neurotological evaluation including Balance Rehabilitation Unit posturography.

**Results:**

The stability threshold mean values were significantly lower (*p* < 0.0001) in users of illicit drugs, with or without alcohol abuse when compared to the control group; the mean values for sway velocity and ellipse area in all evaluated conditions were significantly higher (*p* <0.05) in the experimental group when compared to the control group, except for the ellipse area in static force surface and opened eyes (*p* = 0.168).

**Conclusion:**

The balance control of individuals addicted to illicit drugs with or without alcohol abuse could present stability threshold, sway velocity and ellipse area abnormalities in static posturography.

## INTRODUCTION

Ototoxic agents, such as illegal drugs and alcohol, may cause dizziness^1^.

Psychotropic drugs have different mechanisms of action, and may be classified into groups, including the stimulants (cocaine, amphetamines, etc.), opiates and opioids (heroin, methadone, etc.), sedatives (benzodiazepines) and numerous drugs, including ketamine and cannabis (marijuana)^2^.

Cocaine is the second most frequently used illicit drug, second to *cannabis*, in the United States and Europe, it still is the psycho-stimulant of choice for many, often times mixed with other psychotropic substances. It is frequently used with alcohol, and the alcohol abuse diagnosis is made in 50% to 90% of the subjects addicted to cocaine^3^.

In one study involving the 108 largest cities in Brazil, marijuana, cocaine and *crack* use were prevalent among males of all age ranges; people with more than 35 years of age had the highest percentage of cases submitted to treatment, reaching the figure of 6.2%^4^.

Alcohol may affect central nervous system structures which regulate the oculomotor and body balance systems, including the central vestibular system, vestibular nuclei and the cerebellum^5^^,^^6^.

Since body balance results from the integration of visual, vestibular and proprioceptive afferent stimuli, its deficits may be identified by means of patient exposure to different sensorial situations upon posturography. This method of postural stability assessment may be static or dynamic, on situations which bring stress to posture control and assess the patient's ability to maintain balance using only one given sensory clue, reducing or changing the support base, or making head movements^7^. Static posturography with the *Balance Rehabilitation Unit's* (BRU^TM^) virtual reality was designed to reproduce, under different somatosensory situations, the visual, vestibular and visual-vestibular stimuli to which the patient is exposed in his daily life, supplying information about the position in the pressure center under ten sensory situations, by means of quantitative indicators: stability threshold area, pressure center shifting area (ellipse area) and sway velocity^8^.

Upon static posturography in healthy individuals, body sway with the eyes open and with the eyes closed had a positive correlation with the concentration of alcohol in the blood one hour after drinking whiskey^4^^,^^5^. Nonetheless, after an acute intake of alcohol, there was an increase in the sway area and in cross-sectional sway in the low frequencies (0-1 Hz), especially with the eyes closed, without any correlation with the alcohol in the blood^9^.

Upon dynamic posturography, in healthy individuals who drank 0.6 g/Kg of alcohol, significant effects of such substance were seen upon body balance in the fixed and in the unstable platforms, with the eyes closed; and findings were near significance with the platform and unstable environments^10^. The effects of acute intoxication were found in the platform which moves in sync with the patient's anteroposterior sway with the eyes open and closed, suggesting a disorder in the integration among vestibular and visual information or reduction in the vestibular function^11^.

In healthy individuals with low blood alcohol content (0.45 g/Kg); average content (0.80 g/Kg) and high (1.05 g/Kg), and dynamic posturography showed a high sensitivity, especially considering situations in which the proprioceptive clues were distorted and the visual clues were absent or distorted^12^.

Chronic alcoholics, in variable withdrawal periods varying between one and 20 years, showed lower balance indexes than those found in the control group under all sensorial situations in the dynamic posturography^13^.

Upon dynamic posturography, the dose of 3.3% of tetrahydrocannabinol (THC) - the psychoactive compound of marijuana, with or without alcohol ingestion, increased significantly body sway; and the combination of alcohol and marijuana produced an effect which was similar to that of marijuana alone^14^.

Comparing the dynamic posturography from 10 ex-alcoholics, 10 ex-alcoholics and users of illicit drugs and 10 individuals in the control group, we found clues that the drugs influence more the proprioceptive system, and alcohol may affect the vestibular system, besides the proprioceptive one^15^.

Since the prevalence of drug addicts is increasing^16^, we need further studies on the effects and consequences illicit agents have on the human body; especially on the influence of abuse on the labyrinth and on the mechanisms which maintain body balance.

We did not find references as to the use of static posturography in the *Balance Rehabilitation Unit* (BRU^TM^) to assess the body balance of illicit drug users, which was the reason to carry out this study.

The goal of the present study is to assess body balance with static posturography in the *Balance Rehabilitation Unit* (BRU^TM^), in drug users, with or without alcohol abuse.

## METHOD

This study was carried out after having been approved by the Ethics in Research Committee of the Institution, under protocol # 1548/07. All participants signed the Informed Consent Form before we started the investigation.

In this controlled and cross-sectional study, the experimental group was made up of 47 male individuals, aged between 25 and 55 years, addicts to illicit drugs, with or without alcohol abuse, who were in the Reference Center for Alcohol, Tobacco and Other Drugs (CRATOD), São Paulo, Capital, at the time when the present study was carried out.

The control group was made up of 47 healthy individuals from the university community where the study was carried out, paired by gender and age, without otological complaints and without a past of illicit drug or psychoactive medication, who occasionally drank small amounts of alcohol and who had not taken alcohol within 24 hours before the exam.

As inclusion criterion, experimental group individuals should have a diagnosis of being addicted to marijuana, cocaine and/or *crack*, according to the DSM-IV^17^ (*Diagnostic and Statistical Manual of Mental Disorders)* either with or without alcohol abuse, without neurological signs and with a good nutritional status, characterized upon medical assessment. We included those individuals who used psychiatric medication i.e. tranquilizers, antidepressants and anti-seizure medication, since breaking the use of this medication would compromise the patient and his/her treatment in our institution.

We took off the study those patients who had not used drugs for more than 30 days, those who reported drinking alcohol or used illicit drugs for at least 24 hours prior to the assessment; unable to understand simple verbal orders; unable to remain alone standing up; with severe visual impairment or not compensated with corrective lenses; and those with neurological and orthopedic disorders which resulted in movement limitation or the use of prosthesis in the lower limbs were also taken off the study. In order to maintain the group's homogeneity as far as gender is concerned, we took off the females, because most of the patients under treatment at the CRATOD, at the time when this study was carried out were males.

Individuals from the experimental and control groups were submitted to an interview, with the aim of checking the occurrence of symptoms associated with the vestibular system, and identify the types of drugs used, alcohol intake, use of medication, duration and amount of the drug used; ear canal inspection looking for ear wax and/or a foreign body in the external acoustic canal; vestibular function assessment^18^^,^^19^; based on positional and positioning nystagmus exam and computerized vector electronystagmography, including a study of spontaneous nystagmus with the eyes open and closed; semi-spontaneous nystagmus; saccadic movements; pendular tracking; optokinetic nystagmus, pre and per-rotational nystagmus; and, pre and post-caloric nystagmus, and *Balance Rehabilitation Unit* (BRU^TM^)^20^^,^^21^ posturography.

The *Balance Rehabilitation Unit* (BRU^TM^)^20^ posturography, carried out with the patient standing up, with this arms hanging next to the body, identified the pressure center position by means of the upper stability threshold, body shifting in the anteroposterior direction and lateral using the ankle strategy, without moving one's feet or resort to trunk strategies; and the ellipsis area and sway velocity under ten sensory situations: 1) the ellipsis area and sway velocity in ten sensory situations: 1) on a hard surface and with the eyes open, 2) on a hard surface and eyes closed, 3) on a foam pillow and eyes closed, 4) on a hard surface and saccadic stimulation, 5) on a hard surface and optokinetic stimulation in the horizontal direction from left to right, 6) on a hard surface and optokinetic stimulation in the horizontal direction from right to left, 7) on a hard surface and optokinetic stimulation in the top-down vertical direction, 8) on a hard surface and optokinetic stimulation in the bottom-up vertical direction, 9) on a hard surface and horizontal stimulation association with slow and uniform head rotation movements, 10) on a hard surface and optokinetic stimulation in the vertical direction, associated with slow and uniform flexion and extension movements of the head.

We carried out a descriptive statistical analysis in order to characterize the sample.

We used the *t-Student* test in the comparative analysis of the groups (experimental group of illicit drug users with or without alcohol abuse and controls) concerning the variables: age, stability threshold, sway velocity and elliptical area under the BRU^TM^ situations. Variables age, sway velocity and ellipsis area were transformed by means of a logarithmic function because assumption of normality was rejected by the *Kolmogorov-Smirnov* test. The stability threshold variable did not change. The test power was calculated in the tests in which there was a significant difference between the groups. The values we found varied between 62.5% and 100.0%, showing that sample size was enough to obtain approximately 80% of power.

The *t-Student* test was carried out in the comparative analysis involving the experimental group with medication and without medication, and in the analysis of variables: stability threshold, sway velocity and ellipsis area under BRU^TM^. Variables ellipsis area on a hard surface with the eyes open and closed, foam on closed eyes, optokinetic stimulation to the right, to the left, up and down, horizontal and vertical visual-vestibular interaction and sway velocity under optokinetic stimulation to the right, downwards and upwards were transformed by means of a logarithm function, for not having a normal distribution. In order to study the correlation between the duration of illicit drug use, in years, and variables: stability threshold, sway velocity and ellipse area under the BRU™ we used the Pearson's correlation coefficient.

The analyses were carried out by the SPSS 10.0 software for Windows, and the significance level established for the statistical tests was 5% (α = 0.05).

## RESULTS

We assessed 47 male users of illicit drugs, with or without alcohol abuse in the control group. As far as age is concerned, the experimental group had a mean age of 38.30 ± 8.03 years (varying between 25 and 55 years) and the control group had mean age of 36.87 ± 8.71 years (varying between 25 and 54 years). We did not find significant difference between the groups in relation to the mean age (*p* = 0.411).

Marijuana abuse was diagnosed in 22 (46.8%) users, cocaine in 39 (83%) and *crack* in 34 (72.3%). Of the 47 cases, 40 (85.1%) abused alcohol. The mean duration of drug use was 17.32 years (standard deviation = 10.52) and the mean duration of alcohol use was 22.38 years (standard deviation = 9.64).

Of the 47 cases, 32 (68.1%) were being treated with one or more medications, such as diazepam, carbamazepine, clonazepam, phenobarbital, naltrexone, fluoxetine, lithium carbonate, bromazepam and haloperidol; and 15 (31.9%) were not using any medication.

As to clinical history, 27 (57.4%) users of illicit drugs with or without alcohol abuse reported dizziness and 19 (40.4%) reported balance disorders. Dizziness and/or balance disorder were reported by 22 of the 32 cases (68.8%) with medication prescription and by eight of the 15 (53.3%) without medication.

Upon assessment of the vestibular function in investigating the positioning, positional, spontaneous, semi-spontaneous and optokinetic nystagmus; pendular tracking, decreasing pendular rotary test, and caloric test, 43 cases of illicit drug users (91.5%) had results within normal ranges; and four (8.5%) had post-caloric nystagmus hyporeflexia, as sole finding, indicating peripheral vestibular dysfunction.

Upon the *Balance Rehabilitation Unit* (BRU^TM^) posturography, there was a statistically significant difference (*p* < 0.0001) between the values of the stability threshold area (cm^2^) of the control group (mean = 242.94; standard deviation = 64.51; median = 244.00; variation = 113-469) and the values from the group of illicit drug users, with or without alcohol abuse (mean = 191.74; standard deviation = 60.20; median = 195.00; variation = 89-336). The mean values of the stability threshold area from the group of illicit drug users with or without alcohol abuse were lower than those from the control. Concerning the group of illicit drug users, with or without alcohol abuse, there were no statistically significant differences (*p* = 0.553) between the values of the stability threshold area (cm^2^) from the group with medication (mean = 188.13; standard deviation = 63.41; median = 191.00; variation = 89-336) and the values from the group without medication (mean = 199.47; standard deviation = 53.96; median = 203.00; variation = 118-299).

Table 1 shows the sway velocity (cm/s) descriptive values and comparative analysis in the control groups and illicit drug users, with or without alcohol abuse. The sway velocity and ellipsis area mean values from the illicit drug users group with or without alcohol abuse were higher than the control group in all the situations assessed, with a statistically significant difference (*p* < 0.05), except for the first ellipsis area situation, on a hard surface and with the eyes open (*p* = 0.168).

**Table 1:** Descriptive values and comparative analysis of the sway velocity (cm/s) and ellipsis area (cm^2^) in the *Balance Rehabili tation Unit* (BRU™) situations of 47 individuals from the control group and 47 users of illicit drugs, with and without alcohol abuse.

		Sway velocity			Ellipsis area		
		Mean	SD	p-value	Mean	SD	*p*-value
	Control	0.69	0.23	0.025*	1.75	1.10	0.168
HS/Open eyes/without stimulus	Drugs	0.81	0.31		2.84	2.67	
	Control	0.95	0.32	0.025*	1.84	1.47	0.009*
HS/Closed eyes	Drugs	1.12	0.40		4.37	6.22	
	Control	2.15	0.70	0.003*	5.76	2.81	< 0.001*
Foam/Closed eyes	Drugs	2.76	1.15		12.30	8.73	
	Control	0.89	0.26		1.33	0.84	
HS/Saccadic	Drugs	1.17	0.37	< 0.001*	2.77	2.60	0.001*
	Control	0.84	0.30	0.002*	1.44	1.08	< 0.001*
HS/Optokinetic to the right	Drugs	1.11	0.54		5.96	12.65	
	Control	0.85	0.32	0.001*	1.31	1.04	< 0.001*
HS/Optokinetic to the left	Drugs	1.14	0.52		4.96	10.48	
	Control	0.94	0.45	0.009*	1.80	1.62	0.002*
HS/Optokinetic upwards	Drugs	1.16	0.51		5.67	11.90	
	Control	0.92	0.42	< 0.001*	1.55	1.54	< 0.001*
HS/Optokinetic downwards	Drugs	1.28	0.65		6.33	13.89	
	Control	1.27	0.54	< 0.001*	2.71	2.53	< 0.001*
HS/Horizontal Visual-Vestibular Interaction	Drugs	1.68	0.62		6.11	7.45	
	Control	1.47	0.48	< 0.001*	2.62	1.55	< 0.001*
HS/Vertical Visual-Vestibular Interaction	Drugs	2.11	0.62		7.12	7.68	

* Significant values; HS: hard surface; SD: standard deviation; *t-Student test;* significance level α 0.05.

Table 2 shows the descriptive values and the comparative analysis vis-à-vis the sway velocity (cm/s) and the ellipsis area (cm^2^), from the group of illicit drug users, with or without alcohol abuse, in relation to using or not using medication. There was not significant difference vis-à-vis the mean values of the sway velocity and the ellipsis area between the group which used or didn't use medication.

**Table 2:** Descriptive values and comparative analysis of the sway velocity (cm/s) and the ellipsis area (cm^2^) of the *Balance Reha bilitation Unit* (BRU™) conditions of 47 users of illicit drugs, with and without alcohol abuse, with and without medication.

		Sway velocity			Ellipsis area		
	Groups	Mean	SD	*p*-value	Mean	SD	*p*-value
	W/medication	0.81	0.33	0.847	3.11	3.03	0.858
FS/Eyes Open/Without Stimuli	W/out medication	0.82	0.27		2.25	1.51	
	W/medication	1.12	0.41	0.841	4.78	7.00	0.860
FS/Eyes Closed	W/out medication	1.14	0.40		3.51	4.17	
	W/medication	2.82	1.27	0.575	12.90	9.41	0.379
Foam/Eyes Closed	W/out medication	2.62	0.85		11.02	7.19	
	W/medication	0.17	0.40		2.88	2.90	
FS/Saccadic	W/out medication	1.15	0.32	0.836	2.56	1.89	0.704
	W/medication	1.14	0.56	0.599	6.27	14.34	0.995
FS/Optokinetic to the right	W/out medication	1.05	0.50		5.31	8.36	
	W/medication	1.18	0.58	0.391	5.80	12.49	0.603
FS/Optokinetic to the left	W/out medication	1.04	0.35		3.17	3.21	
	W/medication	1.15	0.48	0.974	6.26	14.06	0.757
FS/Optokinetic upwards	W/out medication	1.17	0.58		4.41	5.03	
	W/medication	1.29	0.70	0.883	6.98	16.45	0.997
FS/Optokinetic downwards	W/out medication	1.24	0.54		4.93	5.63	
	W/medication	1.67	0.67		6.61	8.53	
FS/Visual-Vestibular/Horizontal interaction	W/out medication	1.60	0.53	0.934	5.02	4.38	0.798
	W/medication	2.07	0.59		6.92	7.48	
FS/Visual-Vestibular/Vertical Interaction	W/out medication	2.19	0.71	0.550	7.56	8.34	0.613

FS: firm surface; SD: standard deviation; *t-Student test;* level of significance α = 0.05.

Duration of illicit drug use, with or without alcohol abuse, had a positive correlation with the sway velocity (cm/s) with foam and eyes closed (*p* < 0.05).

## DISCUSSION

We assessed 47 users of illicit drugs, with or without alcohol abuse, of males between 25 and 55 years of age, under treatment.

Complaints which are directly or indirectly associated with the vestibular system, with dizziness and unbalance, were reported by most users of drugs, with or without alcohol abuse, in contrast to what has been mentioned in another study^22^, which did not find vestibular dysfunction symptoms in users of inhaled cocaine.

Dizziness and/or unbalance was similar in the cases with medical prescription (68.8%) and without medication (53.3%), suggesting that illicit drugs use, with or without alcohol abuse, would have triggered these symptoms, and not the drug treatment instated.

Posturography showed that the stability threshold area values from the group of illicit drug users, with or without alcohol abuse, were lower when compared to the control group, indicating that the ability to maintain balance when the individual moves the body mass center without changing the support base is compromised.

Upon *Balance Rehabilitation Unit* (BRU^TM^) posturography, the group of illicit drug users, with or without alcohol abuse, had sway velocity and ellipsis area values higher than those in the control group in all situations assessed, except for the values of the ellipsis area in the first situation, in other words, on a hard surface and with the eyes open, suggesting dependence on vision to maintain balance and the harmful influence of visual, vestibular and visual-vestibular stimuli on posture control. Notwithstanding, upon dynamic posturography, former users of illicit drugs and alcohol had reduced values with their eyes open on foam only^13^. We did not find literature reports on changes in sway velocity and ellipsis area in users of illicit drugs, with or without alcohol abuse, upon the *Balance Rehabilitation Unit* (BRU^TM^) posturography.

In the *Balance Rehabilitation Unit* (BRU^TM^) posturography, the stability threshold area, sway velocity and ellipsis area value of the drug users, with or without alcohol abuse, under treatment with numerous medications, were similar to those in the group of illicit drugs, with or without alcohol abuse without using medication, indicating that there was no influence of the medication on the posturography results. Therefore, the abnormal values hereby found may have stemmed from using illicit drugs, with or without alcohol abuse, regardless of medication use.

The duration of illicit drug use was positively correlated with the sway velocity with foam and eyes closed. Thus, the longer the time of illicit drug use, the greater the difficulty for the individual to maintain body balance when the visual and proprioceptive clues are missing or distorted.

Our results indicated that static posturography provides relevant information on the body balance of patients who abuse illicit drugs, with or without alcohol abuse, and helps identify the impaired sensory situations, associated with visual, vestibular and visual-vestibular interaction stimuli, which should be the target of treatment.

It would be interesting if new studies carried out in this area investigated different stages of intoxication, in an attempt to better understand the influence of illicit drugs and alcohol on body balance.

## CONCLUSION

The body balance of illicit drug users, with or without alcohol abuse, may have abnormalities in the stability threshold area, sway velocity and ellipsis area values associated to visual, vestibular and visual-vestibular interaction stimuli upon the *Balance Rehabilitation Unit* (BRU^TM^) static posturography.

## References

[1] Silva MLG, Munhoz MSL, Ganança MM, Caovilla HH., Silva MLG, Munhoz MSL, Ganança MM, Caovilla HH. (2000).

[2] Milroy CM, Parai JL. (2011). The histopathology of drugs of abuse. Histopathology..

[3] Lacoste J, Pedreira-Melgire M, Charles-Nicolas A, Ballon N. (2010). Cocaine and alcohol: a risky association. Presse Med..

[4] Carlini EA, Galduróz JC, Noto AR, Fonseca AM, Carlini CM, Oliveira LG (2007).

[5] Kubo T, Sakata Y, Matsunaga T, Koshimune A, Sakai S, Ameno K, Ijiri I. (1989). Analysis of body sway pattern after alcohol ingestion in human subjects. Acta Otolaryngol Suppl..

[6] Kubo T, Sakata Y, Koshimune A, Sakai S, Ameno K, Ijiri I. (1990). Positional nystagmus and body sway after alcohol ingestion. Am J Otolaryngol..

[7] Herdman SJ, Tusa RJ., Herdman SJ (2000).

[8] BRU™. Unidade de Reabilitação do Equilíbrio. Manual do usuário. Versão 1.0.7. Versão do Software: 1.3.5.0. Uruguai: Medicaa; 2006. 132p.

[9] Ando S, Iwata T, Ishikawa H, Dakeishi M, Murata K. (2008). Effects of acute alcohol ingestion on neuromotor functions. Neurotoxicology..

[10] Ledin T, Odkvist LM. (1991). Effect of alcohol measured by dynamic posturography. Acta Otolaryngol Suppl..

[11] Tianwu H, Watanabe Y, Asai M, Shimizu K, Takada S, Mizukoshi K. (1995). Effects of alcohol ingestion on vestibular function in postural control. Acta Otolaryngol Suppl..

[12] Goebel JA, Dunham DN, Rohrbaugh JW, Fischel D, Stewart PA. (1995). Dose-related effects of alcohol on dynamic posturography and oculomotor measures. Acta Otolaryngol Suppl..

[13] Ledin T, Odkvist LM. (1991). Abstinent chronic alcoholics investigated by dynamic posturography, ocular smooth pursuit and visual suppression. Acta Otolaryngol..

[14] Liguori A, Gatto CP, Jarrett DB. (2002). Separate and combined effects of marijuana and alcohol on mood, equilibrium and simulated driving. Psychopharmacology..

[15] Mendonça AC, Rossi AG, Flores FT, Teixeira CS. (2006). Alterações do equilíbrio em indivíduos ex-usuários de álcool e drogas ilícitas. Acta ORL..

[16] United Nations Office on Drugs and Crime- UNODC. Global illicit drug trends. 2004. New York: United Nations; 2004.

[17] American Psychiatric Association (1994). Diagnostic and Statistical Manual of Mental Disorders.

[18] Ganança MM, Caovilla HH, Munhoz MSL, Silva MLG, Frazza MM., Caovilla HH, Ganança MM, Munhoz MSL, Silva MLG. (1999).

[19] Ganança MM, Caovilla HH, Ganança FF. (2010). Electronystagmography versus videonystagmography. Braz J Otorhinolaryngol..

[20] Gazolla JM, Dona F, Ganança MM, Suarez H, Ganança FF, Caovilla HH. (2009). Realidade virtual na avaliação e reabilitação dos distúrbios vestibulares. Acta ORL..

[21] Cusin FS, Ganança MM, Ganança FF, Ganança CF, Caovilla HH. (2010). Balance Rehabilitation Unit (BRU^TM^) posturography in Menière's disease. Braz J Otorhinolaryngol..

[22] Taguchi CK, Mitsumori NM, Almeida K, Segarra JA, Cavalli SE, Martins M. (2000). O sistema vestibular e as drogas ilícitas. J Bras Fonoaudiol..

